# Imaging Findings and Management Strategy in Type II Popliteal Artery Entrapment Syndrome: A Tale of Two Cases

**DOI:** 10.7759/cureus.66513

**Published:** 2024-08-09

**Authors:** Resham Singh, Tej Pal, Vineeta Ojha, Sanjeev Kumar

**Affiliations:** 1 Cardiovascular Radiology and Endovascular Interventions, All India Institute of Medical Sciences, New Delhi, IND

**Keywords:** magnetic resonance imaging (mri), paes, intermittent claudication, gastrocnemius, popliteal artery

## Abstract

Popliteal artery entrapment syndrome (PAES) is a frequently underdiagnosed condition that should be investigated in adults who experience exertional intermittent claudication in the lower limbs. If detected early, it is a surgically treatable cause of leg claudication in young individuals. PAES can be inherited or acquired through muscular hypertrophy, and the literature classifies it into six categories (I-VI) based on anatomical type. We intend to report the magnetic resonance imaging (MRI) findings of two cases with type II PAES and their management.

## Introduction

Popliteal artery entrapment syndrome (PAES) is an infrequently described and underdiagnosed vascular compression syndrome of the lower limbs [1]. If detected early, PAES is the most common surgically treatable cause of leg claudication in young patients [2]. Stuart first described this aberrant anatomy around the popliteal fossa in 1879 during cadaveric dissection [3]. The reported incidence of PAES ranges between 0.6% and 3.5% [4]. Nonetheless, it has been estimated that up to 80% of PAES cases are asymptomatic [5]. Bilateral PAES is reported in up to two-thirds of cases [6], and one study suggested a 15:1 male-to-female ratio [1]. PAES can be congenital or acquired through muscle hypertrophy and is widely classified in the literature into six subtypes based on Levien's classification (I-VI) [7]. Among the six subtypes, type II PAES is the most common subtype reported in the literature [2]. Furthermore, the popliteal artery (PA) can be divided into three segments: P1: from the adductor hiatus to the top of the patella; P2: from the top of the patella to the center of the knee joint; and P3: from the center of the knee joint to the top anterior tibial artery (ATA) origin. 

Cross-sectional imaging modalities such as magnetic resonance imaging (MRI) or computed tomography (CT) play a crucial role in assessing anatomic abnormalities in suspected cases of PAES.

We report two cases of bilateral type II PAES presenting with symptomatic PAES on the right side and asymptomatic PAES on the left side. We aim to highlight the importance of cross-sectional imaging in diagnosing aberrant anatomy in type II PAES and individualizing the treatments according to the anatomical status of the artery and its adjacent structures.

## Case presentation

Case 1

**Figure 1:**
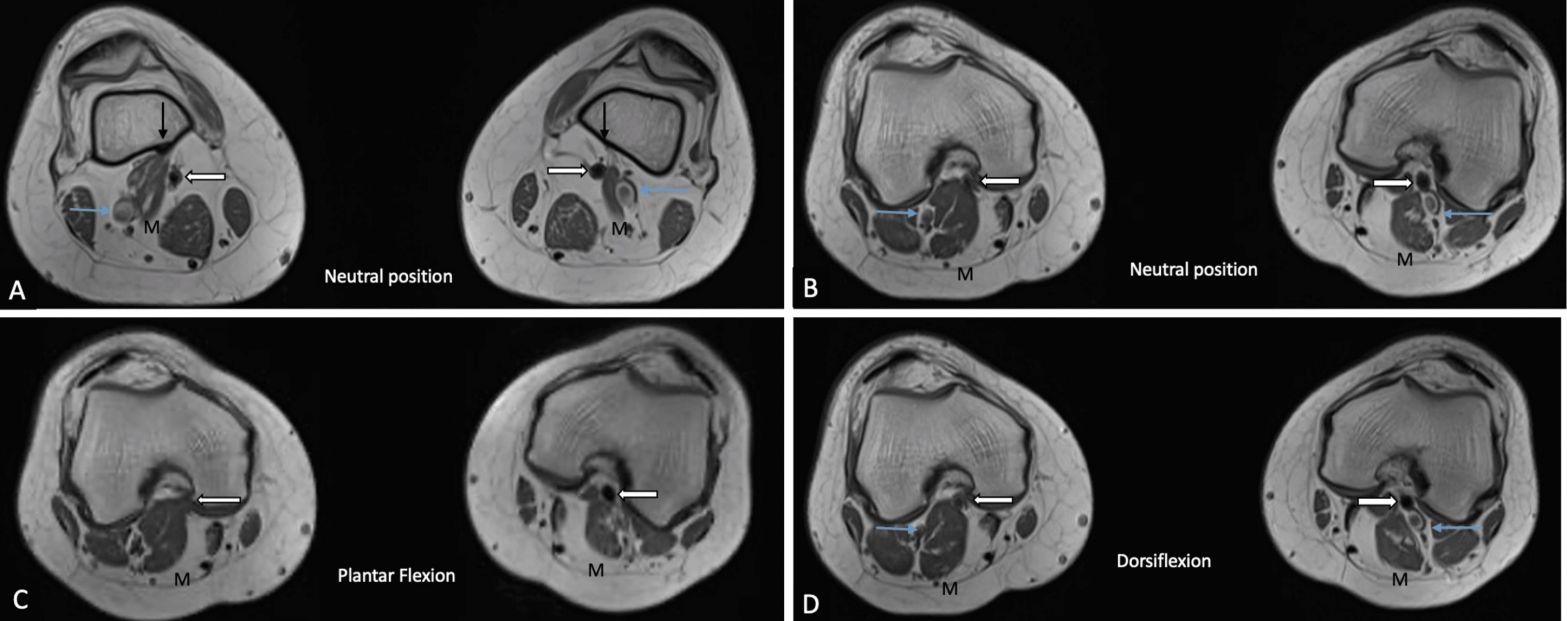
Axial T1-weighted images of the popliteal region in different dynamic maneuvers: 1A and 1B show the neutral position, 1C shows plantar flexion, and 1D shows dorsiflexion. Figure 1A shows an abnormally high and lateral insertion of the medial head of the gastrocnemius (M) just above the lateral aspect of the medial femoral condyle bilaterally (straight black arrow). The muscle belly is seen laterally deviated between the PV (blue arrow) laterally and PA (white arrow) medially, causing significant extrinsic compression of the PA on the right side (white arrow; Figure 1A-1B). On plantar flexion (Figure 1C), there was significantly increased compression of the PA (white arrow) on the right side, and no significant luminal narrowing of the PA (white arrow) was seen on dorsiflexion (Figure 1D). In addition, no significant compression of the left PA (white arrow) was seen on either plantar flexion or dorsiflexion (Figure 1C-1D). PV: popliteal vein; PA: popliteal artery

A male patient in his 20s presented with right lower limb intermittent claudication for two months. A Doppler study revealed an echogenic thrombus in the right PA with monophasic flow in distal vessels. An MRI was done for the further assessment of abnormal musculoskeletal anatomy around the knee joint with suspicion of PAES. Non-contrast MR angiography revealed an approximately 50% focal luminal narrowing of the P3 segment of the PA. The luminal narrowing was increased during dynamic maneuvers (i.e., plantar flexion). There was a superolateral insertion of the medial head of the gastrocnemius on the distal femur above the medial femoral condyle with lateral deviation of the muscle belly between the PA and popliteal vein (PV), which was causing the medial deviation of the PA and crowding of the popliteal fossa with resultant extrinsic compression of the P3 segment of the PA (Figure 1A-1D). There was occlusion of the right ATA with distal reformation by collaterals. In addition, an abnormally high insertion of the medial head of the gastrocnemius was seen on the left side, its muscle belly coursing between the PA and PV. However, no significant luminal narrowing was seen on the left side. The patient was managed with surgical release of the medial head of the gastrocnemius with a reverse saphenous vein graft interposition in the PA. At the two-year follow-up, he was asymptomatic.

Case 2

**Figure 2:**
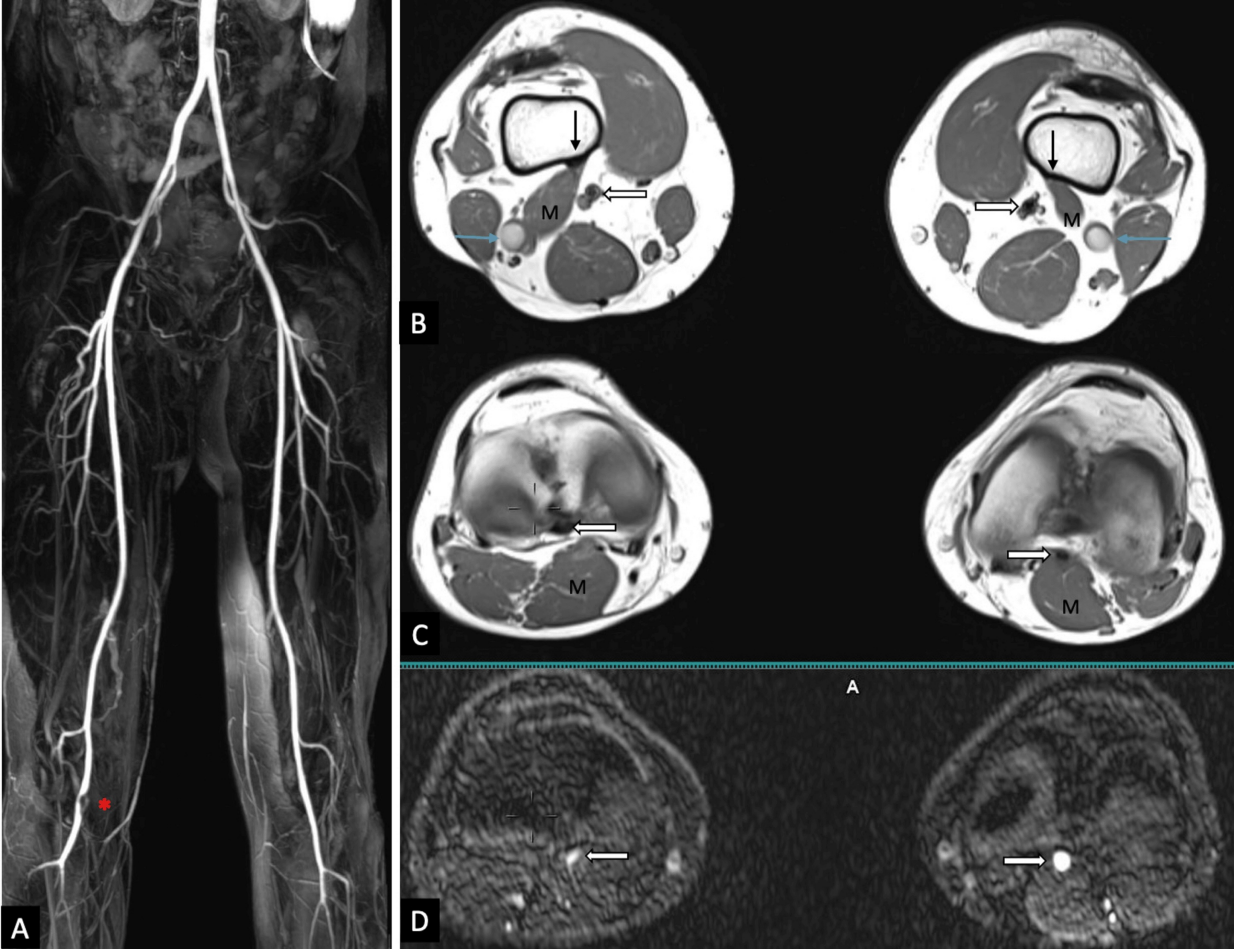
MRI of the popliteal region and non-contrast bilateral lower limb MR angiography of a patient with suspected PAES. Figure 2A's coronal MIP image of non-contrast MR angiography images shows an eccentric filling defect (red asterisk) involving the P2 part of the PA. Axial T1-weighted images (Figure 2B-2C) show an abnormally high and lateral insertion of the medial head of the gastrocnemius (M) just above the lateral aspect of the femoral condyle bilaterally (straight black arrow). The muscle belly is seen laterally deviated between the PV (blue arrow) laterally and the PA (white arrow) medially, causing significant extrinsic compression of the PA on the right side (white arrow; Figure 2B-2D). No significant compression of the PA (white arrow) was seen on the left side (Figure 2B-2D). MRI: magnetic resonance imaging; PAES: popliteal artery entrapment syndrome; MIP: maximum intensity projection; PA: popliteal artery; PV: popliteal vein

A 28-year-old male marathon runner by profession presented with intermittent claudication in the right lower limb for one month. A Doppler study showed an echogenic thrombus in the right PA with monophasic flow in distal vessels. An MRI was done to define the musculoskeletal anatomy further, determine the type of PAE, and plan surgical management. It revealed an approximately 70% focal luminal narrowing of the P2 segment with an eccentric thrombus. There was a lateral insertion of the medial head of the gastrocnemius above the lateral aspect of the medial femoral condyle with hypertrophy of the medial head of the gastrocnemius. There was a lateral deviation of the muscle belly between the PA and PV, which was causing medial displacement and resultant extrinsic compression of the P2 segment of the PA. In addition, an abnormally high lateral insertion of the medial head of the gastrocnemius was seen on the left side, its muscle belly coursing between the PA and PV. However, no extrinsic compression was seen on the left side (Figure 2A-2D). The patient was managed with excision and release of the gastrocnemius muscle band with a reverse saphenous vein interposition graft. At the four-year follow-up, the patient was asymptomatic with no recurrence of symptoms.

Table 1 depicts the clinical details, vascular involvement, management, and follow-up of two cases of type II PAES.

**Table 1:** Clinical details, vascular involvement, management, and follow-up of two cases of type II PAES. PAES: popliteal artery entrapment syndrome; PA: popliteal artery

Case no.	Age (years)	Sex	Profession	Clinical presentation	Vessel involved	Segment of PA affected	Distal thromboembolism	Concomitant vessel involvement	Management	Follow-up
Case 1	21	Male	Medical student	Intermittent right lower limb claudication for two months. Non-smoker	Right PA	P3	Present	Occlusion of the proximal peroneal artery	Surgical release of the medial head of the gastrocnemius with reverse saphenous vein graft interposition	Asymptomatic, at two-year follow-up
Case 2	28	Male	Professional runner	Right lower limb claudication for one month. Non-smoker	Right PA	P2	Present	Occluded mid-anterior tibial artery	Excision and reverse saphenous vein interposition graft with the release of the gastrocnemius band	Asymptomatic, at four-year follow-up

## Discussion

The normal anatomical relationships in the popliteal fossa include the PA and PV, as well as the tibial and common peroneal nerves from the medial to lateral side between the medial and lateral head of the gastrocnemius [1]. In type II PAES, there is more lateral insertion of the medial head of the gastrocnemius than usual and normal descent of the PA, which passes medial to the laterally inserted medial head of the gastrocnemius muscle. The abnormal relation of the PA makes it susceptible to microtrauma, vascular injury, and resultant arterial occlusion [6]. Among all PAES subtypes, type II showed a higher incidence of arterial occlusion than others because there are stronger compressive forces on the PA due to more lateral insertion of the medial head of the gastrocnemius [2].

This abnormal anatomic relationship between the vascular and surrounding musculotendinous tissues causes popliteal entrapment. Furthermore, stenosis and turbulent flow across the entrapped PA may cause post-stenotic ectasia or aneurysm formation. PAES is a progressive condition, so early detection is crucial because early treatment can prevent adverse outcomes such as PA injury, embolism, and lower limb ischemia [8].

Anatomical PAES is diagnosed with high accuracy by MRI using axial T1- and T2-weighted MR images acquired in neutral, plantar flexion, and dorsiflexion (dynamic maneuver) which clearly defines the muscles and anatomical borders of the popliteal fossa. An abnormal lateral insertion of the medial head of the gastrocnemius, medial displacement, blockage of the PA in the popliteal fossa, and adipose tissue occupying the area normally occupied by the medial head of the gastrocnemius are the abnormalities that differentiate anatomical PAES from functional PAES or other causes of claudication in young adults.

Surgical repair of the anatomical anomaly should be considered for type II PAES irrespective of the symptoms' severity, as the natural progression includes arterial damage and occlusion over time. Surgical myotomy of the anomalous gastrocnemius insertion has been performed in type II PAES, with patency rates of up to 96% at one year and 91% at five years [9]. It involves the surgical release of the abnormal musculotendinous slip with the revascularization of the stenosed or occluded popliteal artery using interposition reverse saphenous venous graft.

## Conclusions

PAES is an uncommon cause of claudication in young adults that can be surgically corrected. These two case reports demonstrate the value of MRI with dynamic maneuvers in identifying the type of PAES and accurately depicting the anatomy in the popliteal fossa. The diagnosis of PAES and planning of subsequent treatment can be aided by cross-sectional imaging, which can accurately reflect the anatomic vascular relationship in the popliteal fossa when young people present with intermittent claudication and Doppler findings are suggestive of low monophasic flow in below-the-knee vessels.
